# New opportunities and challenges to engineer disease resistance in cassava, a staple food of African small-holder farmers

**DOI:** 10.1371/journal.ppat.1006287

**Published:** 2017-05-11

**Authors:** Rebecca S. Bart, Nigel J. Taylor

**Affiliations:** Donald Danforth Plant Science Center, St. Louis, Missouri, United States of America; THE SAINSBURY LABORATORY, UNITED KINGDOM

The storage root crop cassava (*Manihot esculenta*) is the most important source of dietary calories in the tropics after rice and maize [1]. It plays a central role in food and economic security for small-holder farmers and holds unrealized potential as a cash and commodity crop. In Africa, cassava production is constrained by three major diseases: cassava mosaic disease (CMD), cassava brown streak disease (CBSD), and cassava bacterial blight (CBB) [2]. Strong inbreeding depression and heterozygosity present challenges to conventional cassava breeding programs. While important advances are being made with genotype-by-sequencing technologies and marker-assisted breeding, these techniques alone will not unlock the crop’s full potential. Historically, cassava has received less research investment than other major crops. Over the last decade, however, multiple genomic platforms and diverse germplasm collections have been developed, along with effective genetic transformation protocols. As a result, numerous reports of genetically engineered cassava have now been published (see Chavarriaga-Aguirre and colleagues) [3]. Here, we briefly summarize previous efforts with an emphasis on technologies relevant to small-holder farmers in Africa. In addition, we discuss new opportunities for using genome-editing technologies to create disease-resistant cassava plants. We conclude by highlighting additional challenges that must be overcome prior to delivering biotechnology-improved cassava varieties to small-holder farmers in Africa.

## Disease impacts on cassava in Africa

**Fig 1:**
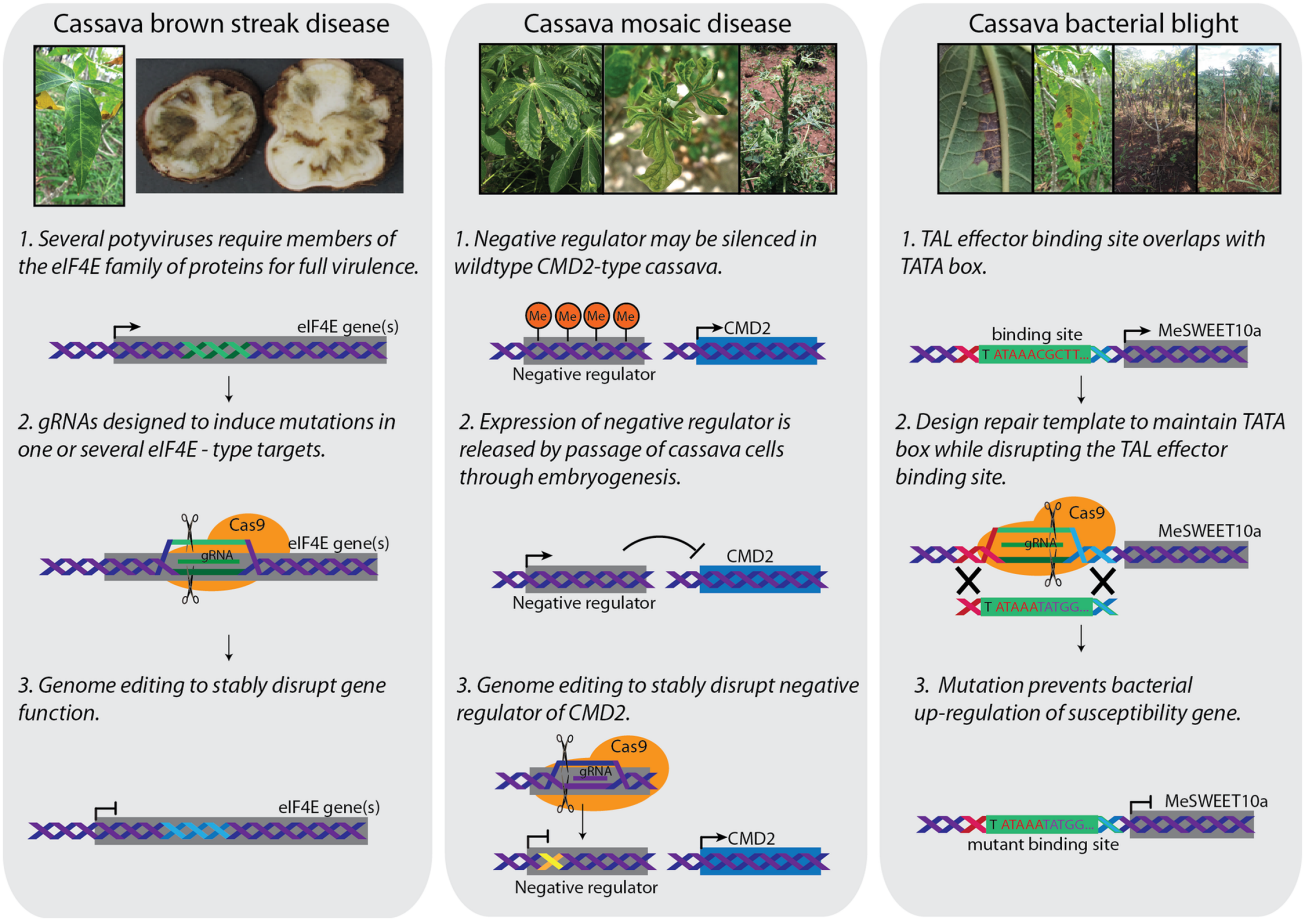
Genome-editing strategies to create resistance against three major pathogens of cassava. (Left) Cassava brown streak disease (CBSD) is caused by two potyviruses. In other species, mutation of eukaryotic translation initiation factor 4E (eIF4E) family of genes promotes disease resistance. It is predicted that a similar strategy may be effective in cassava. (Middle) Cassava mosaic disease (CMD) is widely controlled by the yet to be identified CMD2 resistance mechanism. For unknown reasons, passage of cassava cells through somatic embryogenesis disrupts CMD2-mediated resistance. A potential explanation involves loss of methylation (Me) that releases expression of a negative regulator of CMD2. In this case, it should be possible to use guide RNAs complexed with Cas9 to introduce genetic changes that stably disrupt expression of the negative regulator. (Right) Cassava bacterial blight disease is caused by *Xanthomonas axonopodis* pv. *manihotis* (*Xam*). *Xam* injects transcription activator like (TAL) effectors into plant cells, which activate expression of *MeSWEET10a* by directly binding to its promoter. The binding site overlaps with the TATA box. Using homolog recombination, it would be possible to mutate the binding site, maintain the TATA box, and disrupt TAL effector binding.

The two viral diseases, CMD and CBSD, have a major impact on cassava production in sub-Saharan Africa, together causing estimated losses of US$1 billion per year [4]. A recent report from Kenya estimates US$1,300/hectare losses [5]. Both viruses are transmitted by the whitefly vector *Bemisia tabaci*. CMD is caused by at least seven species of geminiviruses and is endemic across tropical Africa [6]. Infected plants show varying degrees of leaf deformation, mosaic chlorosis, and compromised photosynthetic capacity, leading to reduced storage root yields (Fig 1). Conventional breeding programs have exploited resistance to CMD present within germplasm collections to generate varieties now available to farmers across large parts of Africa [7]. In contrast to CMD, effective control strategies for CBSD are not widely available. Yield loss resulting from CBSD has been described as one of the world’s most important biotic threats to food security [8]. CBSD is caused by two species of potyvirus and results in necrotic lesions within the storage roots, rendering them inedible and unmarketable [9] (Fig 1). In contrast to CMD, no varieties with robust CBSD resistance are available to farmers at this time [8]. Cassava is also susceptible to CBB, caused by the gram-negative bacterial pathogen *Xanthomonas axonopodis* pv. *manihotis* (*Xam*). CBB occurs in Central and South America, across Africa and Asia—everywhere cassava is grown [10]. Disease severity varies from water-soaked lesions on the lower leaves to complete wilting and plant death (Fig 1). It is unknown what triggers periodic severe outbreaks of CBB, although fluctuations in environmental conditions are considered a major factor [3]. To promote disease and suppress host defense responses, *Xam* injects type III effector proteins into host cells. To date, only a subset of these virulence proteins have been studied [11, 12]. As with CBSD, no robust CBB control strategies are available to farmers currently, despite being recognized as an important bacterial pathogen of cassava [13–15].

## Current status of disease-control strategies

### Cassava brown streak disease (CBSD)

Biotechnology provides important and proven potential for engineering CBSD resistance in cassava. RNA interference (RNAi) technology has been shown to generate high-level field resistance to both causal pathogens [16] by transgenic expression of inverted-repeat constructs of the viral coat protein (CP) sequences. Resistance is positively correlated to levels of CP-derived small interfering RNA (siRNA) expression and is capable of reducing losses of usable yield from greater than 95% in the non-modified cultivar to less than 2% in high-expressing transgenic lines [17]. This technology is similar to that deployed successfully to combat Papaya ringspot virus in Hawaii, squash leaf curl virus in cucurbits, and plumpox virus in stone fruits [18, 19]. It offers an important new source of robust resistance to CBSD, which could be made available to farmers and breeders after review and approval by regulatory authorities in East Africa. The advent of CRISPR/Cas9 gene-editing technologies makes an additional CBSD control strategy attractive. Mutation of the eukaryotic initiation factor 4E (eIF4E), or its isoforms, has been demonstrated to generate resistance to the potyviruses that infect diverse species such as *Arabidopsis* spp., pepper, cucumber, and lettuce [20–23]. Application of this technology in cassava (Fig 1) will hopefully generate an additional strategy for the control of this important disease, available for stacking with RNAi and sources of innate resistance.

### Cassava mosaic disease (CMD)

Three types of innate, genetically encoded resistance are exploited for control of CMD. However, in all cases, the underlying molecular mechanisms remain unknown. *CMD1* resistance is thought to be polygenic and recessive, while *CMD2* corresponds to a single, dominant locus on chromosome 12 [24]. Due to its simplicity, *CMD2* is the most widely exploited source of resistance within cassava breeding programs [7, 25]. Recently, a third source of resistance, *CMD3*, was described [26] as consisting of *CMD2* plus an unknown additional factor. A full understanding of these resistance mechanisms at the genetic and molecular levels is required to empower breeders to more effectively generate enhanced planting materials for African farmers. Knowledge of the genetic mechanisms responsible for resistance to CMD would present additional opportunities to engineer enhanced resistance in farmer-preferred varieties of cassava that currently lack CMD resistance. Recently, it was reported that all plants of *CMD2*-type cultivars regenerated through somatic embryogenesis lose resistance to CMD [27]. In contrast, *CMD1*- and *CMD3*-type cultivars maintain resistance through embryogenesis [27]. The discovery that CMD2-mediated resistance is lost by passage of cassava cells through embryogenesis provides previously unavailable, highly similar plant populations that differ in their expression of functional *CMD2*. A unique opportunity now exists, therefore, to unravel what *CMD2* is and how it operates (Fig 1). Once the molecular mechanisms underlying *CMD2* are discovered, transgenic and/or gene-editing technologies can be applied to transfer this trait into new cassava germplasm. It is also possible that some CMD-susceptible cultivars carry non- or partly functional versions of *CMD2*. If so, enticing possibilities exist for genome modification technologies to “activate” *CMD2* in such varieties. Opportunities then arise to combine *CMD2* resistance with *CMD1-* and *CMD3*-type mechanisms to generate stacked, highly durable resistance to all geminiviruses impacting the crop. These endogenous sources of resistance could additionally be stacked with transgenic approaches such as using RNAi to silence viral RNAs [28] and using CRISPR/Cas9 to directly target geminivirus DNA sequences [29].

### Cassava bacterial blight (CBB)

Extensive germplasm collections exist, including wild species of *Manihot* that may harbor sources of resistance to cassava diseases such as CBB. If identified, these traits could be incorporated into cassava breeding programs and/or accessed through biotechnology. In addition to genetically encoded resistance genes, tolerance to pathogens can be achieved by blocking induction of host susceptibility. *Xam* injects transcription activator like (TAL) effectors into cassava leaf cells to induce expression of a sugar transporter (MeSWEET10a), thereby promoting susceptibility [30]. As for rice [31], this discovery opens the door for a genome-editing strategy to block the ability of *Xam* to induce *MeSWEET10a*. Notably the TAL effector binding site within the *MeSWEET10a* promoter overlaps with the TATA box, suggesting that disruption of this sequence might also block endogenous host expression of *MeSWEET10a*. Using homologous recombination, it may be possible to mutate the TAL effector binding site while maintaining the TATA box (Fig 1). An additional challenge remains: if this strategy were deployed in the field, *Xam* TAL effectors would be under strong selective pressure to evolve new binding specificities elsewhere along the *MeSWEET10a* promoter. In addition to *MeSWEET10a*, many additional genes are up-regulated by *Xam* TAL effectors to promote susceptibility [30]. As genome-editing technology continues to be developed, it may become possible to simultaneously disrupt the ability of *Xam* to up-regulate its partial or full complement of susceptibility targets to accomplish a more effective and durable product.

## Challenges for delivering products of new technologies to farmers

Major investments over the previous decade have significantly advanced biotechnological capacities in crops such as cassava. Challenges remain, however, if the resulting products are to reach and benefit small-holder farmers. Some are biological, but others are associated with regulation, approval, and perception of crops enhanced through the use of biotechnology.

### (1) Development/identification of additional sources of resistance

Durable resistance to pathogens can be achieved by stacking multiple sources of resistance. Unfortunately, for most cassava diseases, at best only a single strategy is presently available. Germplasm collections must be more effectively screened to identify additional resistance loci. Once identified, these loci can be transferred among cassava varieties through marker-assisted breeding or biotechnology. Additional research into the molecular mechanisms used by these pathogens and the whitefly vector to cause disease is required and should be prioritized. Such studies are facilitated by genomic resources that are now available for cassava [25, 32–38]. The challenge for leveraging existing and future resources for engineering disease resistance (and other important traits) is two-fold: effective integration of diverse tools, including genomic, transcriptomic, and epigenomic datasets for diverse varieties of cassava, and development of user-friendly platforms to allow scientists with different backgrounds to make use of these resources are needed. Development of such optimized platforms requires targeted investment combined with effective dialogue and creativity among the broader cassava research community.

### (2) Challenges associated with genome editing applied to clonally propagated crops

Genetic transformation protocols for cassava are in place, allowing high-frequency recovery of transgenic plant lines across a range of varieties [39–41]. These tissue culture systems are now being adapted for regeneration of gene-edited events. CRISPR/Cas9 technology has proven effective in diverse crops. However, heterozygous, vegetatively propagated crops such as cassava present additional challenges because removal of integrated genome-editing reagents through sexual crossing causes loss of the desired characteristics within the resulting progeny [36]. Additional research should be prioritized, therefore, on achieving gene editing without integration of the editing reagents. Recent reports in wheat and other species generate optimism that this can be achieved at effective frequencies [42].

### (3) Clarity in the regulatory landscape

The challenges described above present hurdles for the application of biotechnology to generate disease-resistant cassava. There is little doubt that the scientific and technical capacities exist to overcome these—in some cases, within just a few years. Technical success in generating disease resistance in cassava has little meaning, however, if the products are not deployed to farmers. The under-developed regulatory systems in Africa present a significant challenge to delivering the potential of biotechnology to improve small-holder livelihoods. Progress is being made, for example, with recent field trials of cassava for RNAi-mediated resistance to CBSD [16, 17, 43]. However, many countries have yet to enact legislation that would enable deployment of engineered disease-resistant cassava to farmers and breeders. Of growing concern is how products of gene editing will be assessed by African regulatory authorities. It is likely that development of the technologies will outpace the legal regulatory capacities required for timely delivery to end users. It is critical, therefore, that African scientists and regulators are present, active, and influential within ongoing discussions concerning the regulatory status of gene-edited plants. African farmers must not be excluded from benefits that the technologies described here can bring to cassava production.

## Concluding remarks

Recent funding focused on cassava has enabled engagement by an expanding and dedicated group of international plant science researchers. As a result, plant scientists are poised to take advantage of powerful and rapidly developing biotechnologies to engineer disease resistance in cassava and to deliver improved planting materials to African small-holder farmers. In addition to tackling cassava’s weaknesses, an additional noteworthy opportunity exists to use biotechnology to elucidate the molecular and genetic basis of cassava’s drought tolerance and high productivity on marginal lands. This mechanistic insight may then inform efforts to improve the hardiness of other crop species.
